# Lactate dehydrogenase expression modulates longevity and neurodegeneration in *Drosophila melanogaster*

**DOI:** 10.18632/aging.103373

**Published:** 2020-06-02

**Authors:** Dani M. Long, Ariel K. Frame, Patrick N. Reardon, Robert C. Cumming, David A. Hendrix, Doris Kretzschmar, Jadwiga M. Giebultowicz

**Affiliations:** 1 Department of Integrative Biology, Oregon State University, Corvallis, OR 97331, USA; 2 Department of Biology, Western University of London, London N6A 5B7, Ontario, Canada; 3 OSU NMR Facility, Oregon State University, Corvallis, OR 97331, USA; 4 Department of Biochemistry and Biophysics, School of Electrical Engineering and Computer Science, Corvallis, OR 97331, USA; 5 Oregon Institute of Occupational Health Sciences, Oregon Health and Science University, Portland, OR 97239, USA; 6 Present address: Oregon Institute of Occupational Health Sciences, Oregon Health and Science University, Portland, OR 97239, USA

**Keywords:** lactate dehydrogenase, aging, lifespan, lactate, neurodegeneration, circadian rhythms

## Abstract

Lactate dehydrogenase (LDH) catalyzes the conversion of glycolysis-derived pyruvate to lactate. Lactate has been shown to play key roles in brain energetics and memory formation. However, lactate levels are elevated in aging and Alzheimer’s disease patients, and it is not clear whether lactate plays protective or detrimental roles in these contexts. Here we show that *Ldh* transcript levels are elevated and cycle with diurnal rhythm in the heads of aged flies and this is associated with increased LDH protein, enzyme activity, and lactate concentrations. To understand the biological significance of increased *Ldh* gene expression, we genetically manipulated *Ldh* levels in adult neurons or glia. Overexpression of *Ldh* in both cell types caused a significant reduction in lifespan whereas *Ldh* down-regulation resulted in lifespan extension. Moreover, pan-neuronal overexpression of *Ldh* disrupted circadian locomotor activity rhythms and significantly increased brain neurodegeneration. In contrast, reduction of *Ldh* in neurons delayed age-dependent neurodegeneration. Thus, our unbiased genetic approach identified *Ldh* and lactate as potential modulators of aging and longevity in flies.

## INTRODUCTION

Aging is associated with changes in various molecular and cellular processes that ultimately lead to physiological decline and a concomitant increased risk for developing diseases, such as cancer, inflammation, and diabetes [1]. While aging affects all tissues, the brain is especially susceptible to age-related impairments due to its high energy demands. Brain aging is associated with increased mitochondrial dysfunction and dysregulated energy metabolism, which may cause decline in neuronal function leading to neurodegeneration [2, 3].

The causes of age-related brain deterioration have been addressed using a variety of approaches including comparative analysis of gene expression in the brains of young and old organisms. These studies revealed shifts in transcriptional profiles that may underlie age-related alterations in brain function. For example, postmortem analyses of human brain cortex reported significant age-related reductions in genes involved in synaptic plasticity, mitochondrial function, and vesicular transport; and increased expression of genes involved in response to oxidative stress and DNA repair [4]. Similar groups of genes are affected by age in multiple model organisms including *Drosophila* due to the conservation of molecular pathways associated with aging [5]. Most studies examining age-dependent transcriptional changes measured gene expression in samples collected at a single unspecified time of day; however, expression of hundreds of genes change in a daily pattern due to their circadian regulation [6]. Measuring gene expression in samples collected at multiple times of day revealed age-related reprogramming of the diurnal transcriptome in various mammalian tissues [7–9]. Our recent RNA-seq study in *Drosophila* identified hundreds of genes with age-related changes in expression profiles in fly heads [10]. While many genes lost rhythmic expression with age, some genes gain a diurnal rhythmic expression pattern. The most prominent among the latter group was *Ldh* (also known as *ImpL3*) which encodes the metabolic enzyme lactate dehydrogenase. Expression of *Ldh* is low in the heads of young flies; however, it increases several-fold and becomes rhythmic in heads of old flies in light-dark (LD) conditions [10]. Here, we sought to determine whether elevated *Ldh* expression affects aging phenotypes and longevity in flies.

Lactate dehydrogenase (LDH) is an enzyme that catalyzes the conversion of pyruvate, the end product of glycolysis, into lactate (and vice versa) with concomitant interconversion of NADH and NAD^+^. Mammalian LDH proteins are tetramers of subunits encoded mainly by the *LdhA* and *LdhB* genes [11, 12]. The proportion of LDHA and LDHB isoforms composing the tetrameric enzyme complex influences its kinetic and catalytic properties [13, 14]. In *Drosophila*, only one *Ldh* gene is expressed in adults; thus, the LDH enzyme complex exists as a homotetramer of LDH subunits, which share 71 and 75% sequence similarity with the human LDHA and LDHB proteins, respectively [15].

Lactate produced by LDH has long been regarded as a glycolytic waste product; however, many reports suggest that lactate plays important roles in brain energetics. Lactate is considered a glucose sparing metabolite that can fuel neuronal energy production, modulate neuronal excitability, and facilitate memory formation [16, 17]. On the other hand, adverse effects associated with elevated lactate levels have been reported, for example, increased LDH activity and lactate levels have been associated with tumor malignancy [14, 18]. Elevated lactate levels have also been reported in the brains of aged mice [13, 19], Alzheimer’s disease (AD) patients [20], and in the heads of AD model flies [21]. The roles of LDH and lactate in brain functions remain a topic of debate [17, 22–24] and merit further molecular and organismal studies.

Although increased LDH activity and lactate are observed during aging and in AD-related pathologies, it is unknown whether lactate is causally involved in the aging process. To address this question, we studied the biological significance of the age-related increase in *Ldh* gene expression in *Drosophila*. Here, we present evidence that increased *Ldh* expression is associated with elevated LDH protein levels, enzyme activity, and lactate concentrations in the heads of *Drosophila*. Additionally, we show that *in* *vivo* manipulation of *Ldh* gene expression alters lifespan, locomotor activity rhythms, and age-related neurodegeneration in a cell type-specific manner.

## RESULTS

### Aging is associated with increased *Ldh* expression and elevated lactate levels

**Figure 1:**
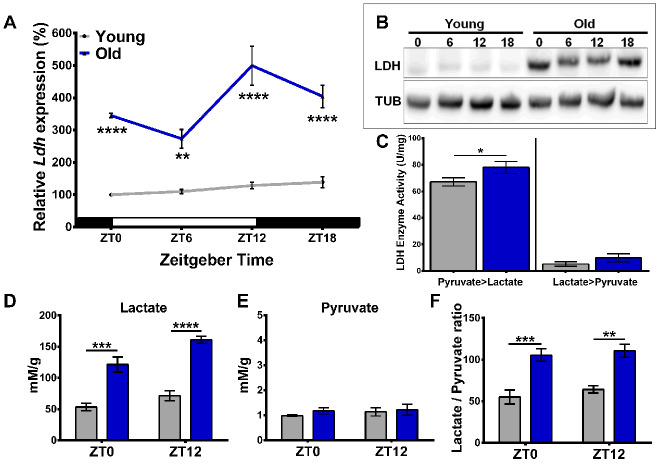
**Age-related increase in *Ldh* expression is associated with elevated LDH protein levels, enzyme activity, and lactate concentration.** (**A**) Profile of *Ldh* mRNA expression in the heads of young (5-days-old) and old (55-days-old) *w^1118^* flies measured by qRT-PCR. Compared to young, old flies have increased *Ldh* mRNA levels at each time point with a peak at ZT12. Values are averages of 4 biorepeats reported as a percentage of expression relative to young at ZT0 set to 100%. (**B**) Representative western blot of LDH protein levels in young and old fly heads with tubulin as a loading control. (**C**) Graph showing LDH enzyme activity for both the pyruvate to lactate and lactate to pyruvate reactions in the heads of young (grey bars) and old (blue bars) flies. Enzymatic activity of LDH is higher in the heads of old flies compared to young for the pyruvate to lactate reaction (*p<0.05 by unpaired one-tailed t-test with Welch’s correction). (**D**) Lactate levels are significantly higher in the heads of old flies (blue bars) at both time points compared to young. In old flies, there is also a significant difference in lactate levels between ZT0 and ZT12 (n=4; p<0.001 by unpaired t-test with Welch’s correction). (**E**) Pyruvate levels do not significantly change between young (grey bars) and old (blue bars) (n=4). (**F**) Average lactate/pyruvate ratios increase in old flies (blue bars) (n=4). Error bars in **A**, **C**–**F** indicate standard error of the mean (SEM). Significance between age groups and time of day in each graph determined by 2-Way ANOVA with Bonferroni's correction (**p<0.01, ***p<0.001, and ****p<0.0001).

To determine the effect of age and time of day on *Ldh* mRNA expression, young (5-days old) and old (55-days old) *white^1118^* (*w^1118^*) males reared in cycles of 12 hours of light and 12 hours of darkness (LD 12:12) were collected every six hours at Zeitgeber time (ZT) 0, 6, 12, and 18. The levels of *Ldh* mRNA were significantly (3-5 fold) higher in the heads of old flies than in young at each time point tested (Figure 1A). Additionally, *Ldh* mRNA levels in the heads of old flies showed a diurnal rhythm with a peak at ZT12 (Figure 1A), in agreement with rhythmicity measured by RNA-seq [10]. To investigate changes in LDH protein levels, we performed immunoblot analysis of head extracts from young and old flies collected at the same time points as samples for *Ldh* mRNA. Due to the lack of a specific antibody against fly LDH, we screened several commercially available LDH antibodies by immunoblotting protein extracts derived from flies with elevated or reduced *Ldh* gene expression. We determined that the antibody against human LDHA (PA5-26531) reacted with fly LDH (Supporting Information Supplementary Figure 1). LDH protein levels were considerably higher in the heads of old flies at each time point, albeit without evidence of significant rhythmic changes (Figure 1B). Since the function of LDH is to catalyze the interconversion of pyruvate and lactate with concurrent changes in the NADH/NAD^+^ redox couple, we next determined LDH enzymatic activity for both reactions by measuring the increase or decrease in NADH over time for each reaction. In both age groups, the activity of LDH was higher for the pyruvate to lactate reaction compared to the conversion of lactate to pyruvate. Notably, the conversion of pyruvate to lactate was significantly increased in the heads of old compared to young, while the conversion of lactate to pyruvate remained similar between age groups (Figure 1C).

Given that LDH protein levels and enzymatic activity of the pyruvate to lactate reaction increase in the heads of old flies, we used 1D ^1^H nuclear magnetic resonance (NMR) to determine whether the concentration of lactate changes in fly heads as a function of age. Polar metabolites were extracted from heads of young and old flies collected at ZT0 and ZT12. Overall, we detected a significant age-dependent increase in lactate concentration at both time points examined (Figure 1D, Supporting Information Supplementary Figure 2). In addition, the level of lactate was significantly higher at ZT12 compared to ZT0 in old flies (Figure 1D) suggesting development of diurnal rhythm during aging, corresponding to the *Ldh* gene expression rhythm (Figure 1A). In contrast to lactate, pyruvate concentrations were not significantly different between the two age groups or time points (Figure 1E). Based on our NMR concentration data, we determined that the lactate to pyruvate ratios were significantly increased in the heads of old flies at both time points (Figure 1F).

### *Ldh* overexpression shortens fly lifespan

**Figure 2:**
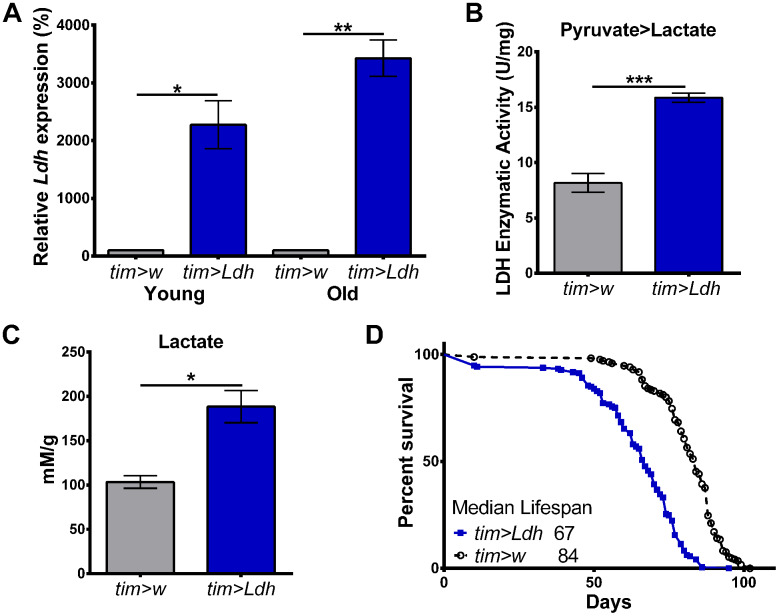
**Flies overexpressing *Ldh* have increased mRNA levels, LDH activity, elevated lactate, and shortened lifespan.** (**A**) *Ldh* mRNA levels were significantly increased in the heads of young and old *tim>Ldh* compared to age-matched *tim>w* control flies. Values are reported as a percentage of expression relative to age-matched *tim>w* set to 100% (n=4). (**B**) LDH enzyme activity was significantly increased for the pyruvate to lactate reaction in the heads of 55-day old *tim>Ldh* flies compared to *tim>w* controls (n=4). (**C**) Lactate concentrations were significantly higher in the heads of *tim>Ldh* flies compared to age-matched *tim*>w controls (n=4). Error bars in A-C indicate SEM. Statistical significance was determined by Unpaired t-test with Welch’s correction (***p<0.001; **p<0.01; *p<0.05). (**D**) Survival curves of *tim>Ldh* (n=193) and *tim>w* (n=170) males. Median lifespan was significantly reduced in *tim>Ldh* flies compared to control (Gehan-Breslow-Wilcoxon test; p<0.0001).

To investigate whether the age-related increase in the expression of *Ldh* mRNA is protective or detrimental during *Drosophila* aging, we measured the lifespan of flies with genetically manipulated *Ldh* expression. Given the rhythmic expression of *Ldh* in old flies (Figure 1A), we first overexpressed *Ldh* in all circadian clock-containing cells using transgenic flies carrying the promoter region of the clock gene *timeless* fused to GAL4 (*tim*-GAL4); these flies were crossed to flies carrying UAS-*Ldh* construct (*tim>Ldh*) or to *w^1118^* flies (*tim>w*) for control. Highly increased *Ldh* mRNA levels were detected in the heads of both 10-day and 55-day old *tim>Ldh* males relative to age-matched *tim>w* control flies (Figure 2A). We also detected significant increases in LDH enzyme activity in the heads of 55-day old *tim>Ldh* flies compared to *tim>w* for the pyruvate to lactate reaction (Figure 2B). Finally, NMR analysis revealed significantly increased lactate levels in the heads of 55-day old *tim>Ldh* flies (Figure 2C). Given that overexpression of *Ldh* increased lactate levels in fly heads compared to age-matched controls, we tested whether the rate of aging is affected in these flies. Overexpression of *Ldh* in all clock cells (a subset of neurons and glia) significantly reduced the median lifespan of *tim>Ldh* males by 17 days (20%) compared to *tim>w* control (Figure 2D).

Since *tim*-GAL4 drives expression in a variety of cell types, including pacemaker neurons, photoreceptors, and most adult glial cells [25, 26], we also evaluated the effects of overexpressing *Ldh* selectively in neurons or glia. *Ldh* is expressed throughout fly development [27]; therefore, we employed the TARGET system [28] to manipulate expression only in adults. Cell-type specific GAL4 driver lines were combined with a *tub*-GAL80^ts^ construct active in all cells, which blocks the action of GAL4 during development at 18°C. After eclosion, the flies were transferred to 27°C or 25°C, which inactivates GAL80^ts^ to varying degrees, allowing GAL4 to induce the expression of the UAS-*Ldh* construct in all clock cells via *tim*-GAL4, all neurons via *elav*-GAL4, or all glia via *repo*-GAL4 (full genotypes are shown in Supporting Information Supplementary Table 1).

**Figure 3:**
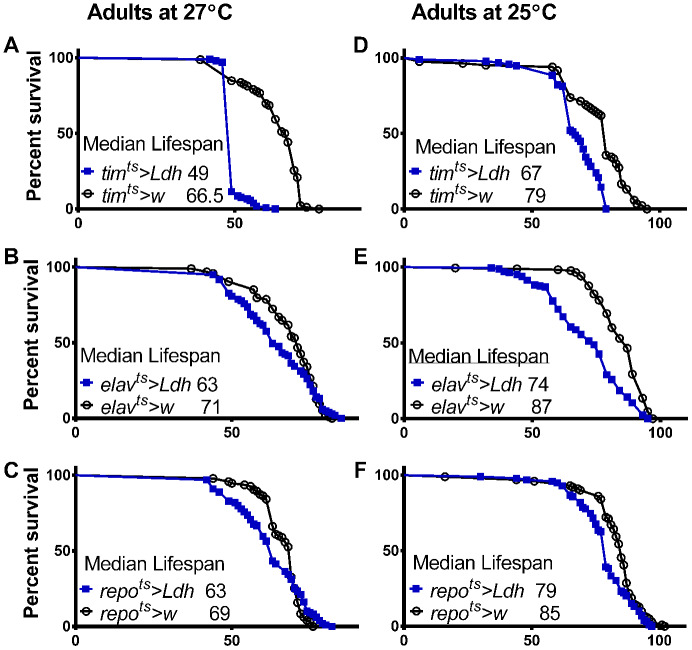
**Adult specific overexpression of *Ldh* shortens fly lifespan.** (**A**–**F**) Survival curves of adult males overexpressing *Ldh* in all clock cells via *tim^ts^* (**A**, **D**), in neurons via *elav^ts^* (**B**, **E**), or in glia via *repo^ts^* (**C**, **F**) each graphed relative to their control group at 27°C and 25°C, respectively. Overexpression of *Ldh* via each driver resulted in decreased median lifespan compared to their respective controls in both 27°C and 25°C (Gehan-Breslow-Wilcoxon test; p<0.05 for graphs B and C and p<0.0001 for graphs A, D, E, and F). See Supporting Information Supplementary Table 2 for experimental details.

Flies overexpressing *Ldh* in all clock cells transferred from 18°C to 27°C after eclosion had significantly reduced median lifespan by 26% compared to *tim^ts^>w* control flies (Figure 3A). A less dramatic but significant lifespan reduction of 11% was observed in *elav^ts^>Ldh* flies with pan-neuronal *Ldh* overexpression compared to *elav^ts^>w* control flies (Figure 3B). Flies overexpressing *Ldh* in all adult glial cells had an 8.5% decrease in median lifespan compared to controls (Figure 3C). Given the abrupt mortality of *tim^ts^>Ldh* flies at 27°C (Figure 3A), we also recorded the lifespan of *tim^ts^>Ldh*, *elav^ts^>Ldh*, *repo^ts^>Ldh*, and the respective control flies transferred to 25°C after eclosion. At 25°C, the lifespan of *tim^ts^>Ldh* flies was also significantly reduced relative to controls (Figure 3D) but less dramatically than in 27°C. Overexpression of *Ldh* in neurons reduced lifespan by 15% while glial overexpression reduced lifespan only by 8% when adult flies were maintained in 25°C (Figure 3E, 3F). The median and maximum lifespan data of all *Ldh* overexpressing flies and controls are provided in Supporting Information Supplementary Table 2.

### Overexpression of *Ldh* in neurons increases neurodegeneration and disrupts circadian locomotor activity rhythms

**Figure 4:**
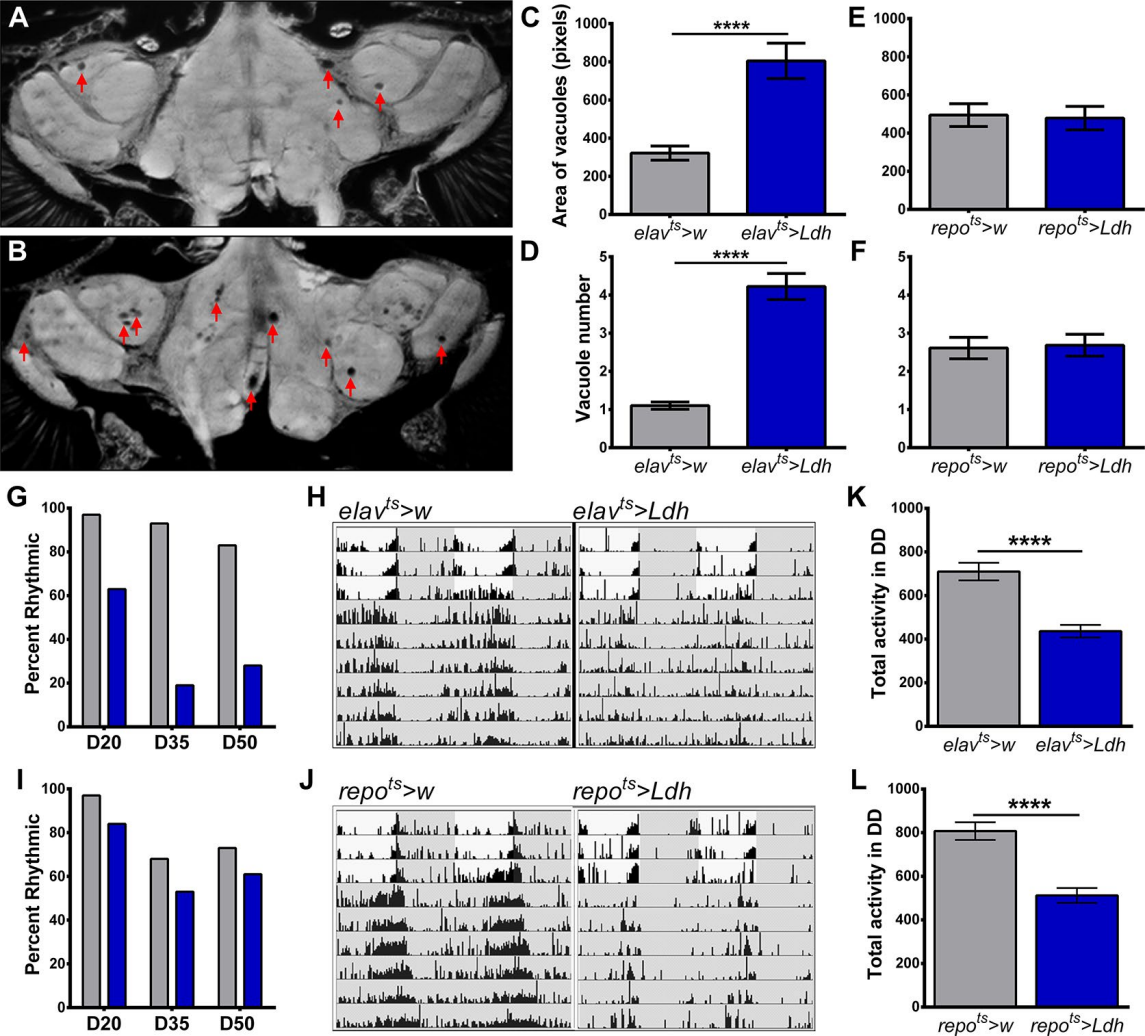
**Flies with neuronal overexpression of *Ldh* show increased neurodegeneration and accelerated decline in locomotor activity rhythms.** (**A**, **B**) Representative brain section images of 55-day old control *elav^ts^>w* (**A**) and *elav^ts^>Ldh* flies (**B**) in 25°C (arrows indicate vacuoles). (**C**, **D**) Graphs show the average area (**C**) and number (**D**) of vacuoles per brain in the brains of *elav^ts^>Ldh* flies and age-matched controls. Both area and the number of vacuoles were significantly increased in *elav^ts^>Ldh* (n=36) flies compared to *elav^ts^>w* (n=38). ****p<0.0001; unpaired t-test with Welch’s correction. (**E**, **F**) There was no significant difference in the average area (**E**) and number (**F**) of vacuoles in 55-day old *repo^ts^>Ldh* (n=19) brains compared to age-matched *repo^ts^>w* control (n=18). Error bars indicate SEM. (**G**) Percent of rhythmic *elav^ts^>Ldh* flies was markedly reduced with age compared to *elav^ts^>w* controls. (**H**) Representative actrograms of individual 50-day old *elav^ts^>w* (rhythmic) and *elav^ts^>Ldh* (arrhythmic) flies. Gray areas indicate lights off. (**I**) Percentage of rhythmic *repo^ts^>Ldh* flies were similar to *repo^ts^>w* controls across lifespan. At least 30 flies were tested for each age group and each genotype. (**J**) Representative actograms of rhythmic 50-day old *repo^ts^>w* and *repo^ts^>Ldh* flies. (**K**) Total daily activity of 35-day old *elav^ts^>Ldh* (n=31) flies averaged over six days in constant darkness was significantly reduced relative to control *elav^ts^>w* (n=30) flies. (**L**) Total daily activity of 35 days-old *repo^ts^>Ldh* (n=30) flies was also significantly lower than in controls (n=31). Statistical significance by unpaired t-test with Welch’s correction (****p<0.0001). Error bars indicate SEM.

We hypothesized that reduced lifespan of flies with elevated *Ldh* gene expression could be linked to brain neurodegeneration. To test this, we evaluated the formation of vacuoles in the brains of *elav^ts^>Ldh*, *repo^ts^>Ldh*, and their respective controls. Vacuoles are a reliable biological marker to measure the extent of neurodegeneration during normal aging or in fly models of neurodegenerative diseases [29–31]. At 55 days of age, the average number and area of vacuoles were significantly higher in *elav^ts^>Ldh* brains than in age-matched *elav^ts^>w* brains (Figure 4A–4D). In contrast to neuronal overexpression of *Ldh*, no significant differences were detected in the number or area of vacuoles in 55-day old *repo^ts^>Ldh* brains compared to *repo^ts^>w* brains (Figure 4E, 4F). Thus, neurodegenerative phenotypes seem to be specifically related to the elevated expression of *Ldh* in neurons, which also causes more dramatic lifespan reduction (see Figure 3E, 3F).

In addition to the increased risk of neurodegeneration, aging is associated with decline in locomotor activity rhythms [32, 33]. Therefore, we monitored the quality of circadian activity rhythms in *elav^ts^>Ldh*, *repo^ts^>Ldh*, and their respective control at 20, 35, and 50 days of age. To assess endogenous circadian activity rhythms, we analyzed the robustness of rhythms over the course of 6 days in constant darkness (DD). Among 20-day old flies, 97% of *elav^ts^>w* control flies and 63% of *elav^ts^>Ldh* flies displayed rhythmic activity (Figure 4G). At 35 and 50 days of age, a high proportion of control flies remained rhythmic while less than 20% of *elav^ts^>Ldh* flies displayed behavioral rhythmicity (Figure 4G, 4H). In contrast, *repo^ts^>Ldh* flies showed only modest decreases in the percentage of rhythmic flies in all age groups (Figure 4I, 4J). In addition to rest/activity rhythms, we tested the effects of *Ldh* overexpression on overall locomotor activity levels by recording the daily locomotor activity counts of 35-day old flies in DD. Compared to their respective controls, the average daily activity counts of both *elav^ts^>Ldh* and *repo^ts^>Ldh* flies were significantly lower (Figure 4K, 4L). Taken together, these data suggest that accelerated loss of locomotor activity is a common feature of *Ldh* overexpression in both neurons and glia. However, neuronal *Ldh* overexpression also accelerates the loss of circadian activity rhythms while *Ldh* overexpression in glia had a modest effect on rhythmicity.

### Reduced *Ldh* expression extends fly lifespan and delays neurodegeneration

**Figure 5:**
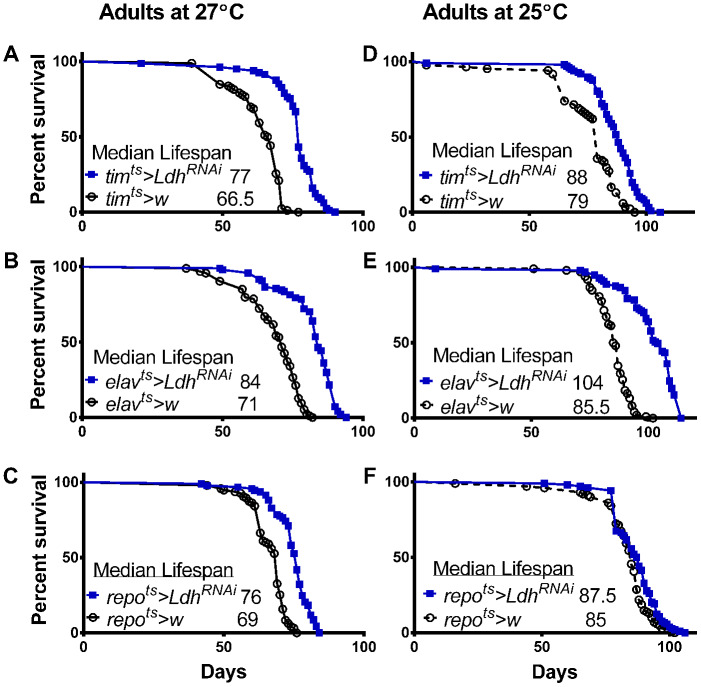
**Decreased *Ldh* expression in adult brain extended lifespan.** Survival curves of males with reduced *Ldh* expression via *Ldh*^RNAi^ combined with *tim^ts^* (**A**, **D**), *elav^ts^* (**B**, **E**), or *repo^ts^* (**C**, **F**) and their controls at 27°C and 25°C, respectively. *Ldh* decrease significantly extends lifespan compared to controls (pairwise comparison by Gehan-Breslow-Wilcoxon test yielded p<0.0001 for all graphs except p<0.05 for *repo^ts^> Ldh*^RNAi^ versus control at 25°C). See Supporting Information Supplementary Table 3 for experimental details.

Data showing that *Ldh* expression at the mRNA and protein level is elevated with age (Figure 1) and that overexpression of *Ldh* reduced lifespan (Figure 3) suggests that *Ldh* is involved in negative regulation of longevity. This hypothesis predicts that decreased *Ldh* expression during aging should enhance longevity. To test this, we used flies with reduced *Ldh* expression in clock cells, neurons, or glia, obtained by crossing the respective *tub*-GAL80^ts^ containing GAL4 lines to flies carrying a UAS-*Ldh^RNAi^* construct. For controls, the same *tub*-GAL80^ts^ were crossed to *w^1118^*. Among flies held in 27°C as adults, lifespan was significantly extended in those with reduced *Ldh* expression in all clock cells, all neurons, or all glia compared to their respective controls (Figure 5A–5C). We also measured survivorship of flies housed at 25°C as adults and observed significant, but moderate increases in median lifespan in *tim^ts^>Ldh^RNAi^* and *repo^ts^>Ldh^RNAi^* flies compared to controls (Figure 5D and F, respectively), while considerable lifespan extension of 21% was recorded in *elav^ts^>Ldh^RNAi^* flies (Figure 5E). The median and maximum lifespan data of all *Ldh^RNAi^* expressing flies and controls are provided in Supporting Information Supplementary Table 3.

**Figure 6:**
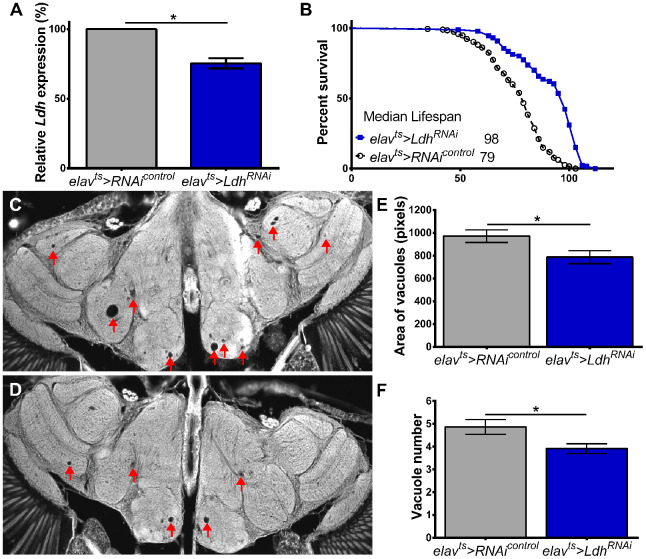
**Neuronal knockdown of *Ldh* delays age-related neurodegeneration.** (**A**) *Ldh* mRNA levels were significantly reduced in the heads of 55-day old *elav^ts^>Ldh^RNAi^* compared to age-matched *elav^ts^>RNAi^control^* flies (n=3; Unpaired t-test with Welch’s correction *p<0.05). (**B**) Survival curves of *elav^ts^>Ldh^RNAi^* (n=187) and *elav^ts^>RNAi^control^* (n=195). Median lifespan was significantly extended in *elav^ts^>Ldh^RNAi^* flies compared to control (Gehan-Breslow-Wilcoxon test; p<0.0001; see also Supplementary Table 3). (**C**, **D**) Representative brain section images of 75-day old (**C**) *elav^ts^>RNAi^control^* and (**D**) *elav^ts^>Ldh^RNAi^* flies (arrowheads indicate vacuoles). (**E**, **F**) Graphs showing that the average area (**E**) and number (**F**) of vacuoles were significantly lower in the brains of 75-day old *elav^ts^>Ldh^RNAi^* flies (n=48) compared to controls (n=46). Statistical significance determined by unpaired t-test with Welch’s correction *p<0.05. Bars indicated SEM.

Given that flies with pan-neuronal knockdown of *Ldh* showed the most substantial lifespan extension, we asked whether reduced *Ldh* expression may have neuroprotective effects. To test this, we evaluated vacuolization in the brains of *elav^ts^>Ldh^RNAi^* flies using as controls progeny of *elav^ts^* flies crossed to UAS-*RNAi^control^* flies, which have the same genetic background as *Ldh^RNAi^* but carry no RNAi construct (Supporting Information Supplementary Table 1). First, we confirmed that the heads of 55-day old *elav^ts^>Ldh^RNAi^* flies had significantly reduced expression of *Ldh* mRNA compared to age-matched *elav^ts^>RNAi^control^* (Figure 6A). Similar to the data shown in Figure 5E, the median lifespan of *elav^ts^>Ldh^RNAi^* flies was significantly increased compared to *elav^ts^>RNAi^control^* flies (Figure 6B, Supporting Information Supplementary Table 3). To account for the extended lifespan of *elav^ts^>Ldh^RNAi^* flies and the fact that 55-day old *elav^ts^>w* control flies show very few vacuoles per brain (Figure 4A); we tested brains of 75-day old flies that show more advanced neurodegeneration to facilitate observing differences between control and experimental flies. Compared to *elav^ts^>RNAi^control^*, the brains of *elav^ts^>Ldh^RNAi^* flies had a significantly smaller number and area of vacuoles (Figure 6C–6F). Taken together, these data suggest that reducing *Ldh* expression in neurons may protect flies from age-associated neurodegeneration.

## DISCUSSION

Recent high-throughput techniques have demonstrated that aging correlates with many changes in gene expression and metabolite levels. However, it is often not clear whether these changes are protective to an aging organism or rather negatively affect healthspan and longevity. In this study, we first show that age-related increase in *Ldh* mRNA and protein levels are associated with elevated lactate levels in the heads of *Drosophila melanogaster*. We then demonstrate that adult-specific overexpression of *Ldh* significantly reduced fly lifespan and that neuronal overexpression increased brain neurodegeneration and accelerated loss of rest/activity rhythms compared to age-matched controls. In contrast, RNAi-mediated decrease in *Ldh* expression, especially in neurons, significantly extended lifespan and showed neuroprotective effects. Together, these data suggest that *Ldh* and lactate play causative roles in the modulation of aging and longevity in flies. We assume that these effects are related to late-life *Ldh* mRNA manipulations; however, because *Ldh* expression was altered through most of adult life, we cannot exclude that chronic changes in LDH enzyme activity could contribute to the observed phenotypes. Likewise, we cannot exclude the possibility that changes in ‘moonlighting’ functions of LDH upon UAS/GAL4 induced *Ldh* overexpression contribute to the phenotypes reported in these flies.

While adult-specific overexpression of *Ldh* in either neurons or glia significantly reduced fly longevity, only pan-neuronal *Ldh* overexpression caused a significant increase in brain neurodegeneration and decay of circadian rest/activity rhythms. This suggests that elevated Ldh levels in neurons have additional detrimental effects. This is supported by the fact that neuronal overexpression of *Ldh* shortened lifespan by 15% while glial overexpression shortened lifespan by only 8%.

We show that age-related increase in *Ldh* mRNA expression results in significantly elevated levels of LDH protein and lactate in fly heads. Given that *Ldh* mRNA is expressed in a diurnal rhythm in the heads of old flies, we measured lactate and pyruvate levels at ZT0 and ZT12. Indeed, in the heads of old flies, lactate levels were significantly higher at ZT12 than at ZT0, which is suggestive of diurnal rhythm. Diurnal rhythmicity in lactate levels have been reported in whole young flies [34], fly bodies [35], and human blood plasma [36]. Our data suggest that putative rhythm in lactate levels is age-dependent in fly heads, in agreement with rhythmic *Ldh* mRNA expression profile detected only in the heads of old flies.

It should be noted that a recent study of age-related metabolic changes in fly heads did not detect an increase in lactate [37]; however, lactate was measured in 35-day old flies and we reported that expression of *Ldh* is only moderately elevated at this age [10]. Our data showing increased lactate levels in the heads of old flies are consistent with the age-related increase in lactate reported in the brain of aging mice [13, 19]. Additionally, elevated lactate levels have been reported in the heads of AD model flies [21] and in an APP/PS1 AD model mice [38]. A significant increase in LDH enzyme activity or lactate was found in the brains of AD patients [20, 39, 40], and increased lactate levels have been suggested as a risk factor in amyloidogenesis related to AD [41]. In another study, elevated levels of lactate negatively correlated with memory performance in humans with mild cognitive impairments [42]. Aging is a well-known risk factor for AD, and it remains to be determined whether age-related increases in LDH and lactate could contribute to the development of AD-related pathologies.

While studies discussed above show links between increased lactate levels and neurodegenerative phenotypes, numerous experiments in young animals or *in vitro* suggest that lactate produced by astrocytes and transferred to neurons may be beneficial for processes that show decline during aging, such as memory formation, neuronal plasticity, and neurogenesis [16, 43]. In addition, a recent report showed that lactate produced by exercising muscles improves glucose tolerance by signaling to adipocytes [44]. Clearly, the reconciliation of reports showing positive and negative effects of lactate will require further investigations. Notably, a recent report suggested that the roles of lactate may change across lifespan as energy metabolism in neurons shifts toward glycolysis in an age-specific fashion [24]. We also note that caution should be used when comparing data obtained in mammals and flies as the ratio of glial cells to neurons increases approximately 6-fold between flies and humans suggesting an expanding role of glia as the complexity of the nervous system increases [45].

Lactate has been proposed as a biomarker of impaired mitochondrial function [42], and normal aging is associated with a decline in mitochondrial quality and activity [3]. Consistent with these metabolic trends, our recent RNA-seq data suggest that age-related increase in *Ldh* expression in fly heads is associated with decline in expression of pyruvate metabolism genes [10]. Following glycolysis, pyruvate is transferred into mitochondria via mitochondrial pyruvate carrier proteins (MPCs) and converted by pyruvate dehydrogenase complex (PDH) to acetyl-CoA, which enters the tricarboxylic acid (TCA) cycle. The activity of PDH is inhibited via phosphorylation by pyruvate dehydrogenase kinase (PDK). Importantly, our recent RNA-seq analysis revealed a significant age-related increase in the expression of the *Pdk* gene in the heads of old flies and a concomitant decrease in expression of the *Mpc1* gene encoding mitochondrial pyruvate carrier [10]. Moreover, two recent transcriptomic studies of the aging fly brain revealed an overall decline in the expression of genes involved in mitochondrial oxidative phosphorylation [46, 47]. Taken together, these data provide evidence of decreased pyruvate uptake by age-damaged mitochondria and/or lowered conversion of pyruvate to acetyl-CoA in addition to decreased mitochondrial oxidative phosphorylation in the aging brain. These mitochondrial deficiencies, when combined with elevated *Ldh* expression, may contribute to increased lactate levels in cells now relying on glycolysis to meet their energetic needs.

Finally, it should be noted that LDH is highly upregulated in many cancers that undergo a metabolic shift towards aerobic glycolysis (Warburg effect) to reduce reactive oxygen species production [14]. This shift includes primary brain tumors; therefore, LDH inhibitors have already been suggested as a potential therapeutic target [14]. Our unbiased genetic approach demonstrates a causative relation between reduced neuronal *Ldh* gene expression and delayed neurodegeneration. Given the conservation of brain aging mechanisms from *Drosophila* to humans, our study highlights the possibility that interventions that reduce LDH activity and lactate levels could conceivably have neuroprotective effects in aging humans.

## MATERIALS AND METHODS

### Fly rearing and genetics

*Drosophila melanogaster* were maintained on a diet containing yeast (35g/L), cornmeal (50g/L), and molasses (5%). Flies were maintained in a 12:12 hour light/dark cycles at 25°C. All experiments were performed on mated adult male flies. We used *w^1118^* (BDSC #5905) flies to investigate changes in *Ldh* mRNA, LDH protein, enzyme activity, and polar metabolites in the heads of young (5 days) and old (55 days) flies. To manipulate the expression of *Ldh* in specific cell types, the *Drosophila* binary UAS/GAL4 system was used. *tim>Ldh* progeny were obtained by crossing *tim*-GAL4 [25] (gift from Jeff Hall) females to UAS-*Ldh* (FlyORF #F002924) males. Control flies were obtained by crossing *tim*-GAL4 females to *w^1118^* males (*tim>w*). To increase spatiotemporal control and manipulate *Ldh* only in adults, we utilized the TARGET system [28]. Temperature-sensitive lines were used to drive expression in all circadian clock cells (*tim*-GAL4;*tub*-GAL80^ts^; gift from Patrick Emery), in all neurons (*elav*-GAL4;*tub*-GAL80^ts^), or all glia (*tub*-GAL80^ts^;*repo*-GAL4; gift from Rob Jackson); we refer to these lines as *tim^ts^*, *elav^ts^*, and *repo^ts^*, respectively. Females carrying these temperature-sensitive drivers were crossed to UAS-*Ldh* males to obtain *tim^ts^*>*Ldh*, *elav^ts^*>*Ldh*, and *repo^ts^*>*Ldh* flies (see Supporting Information Supplementary Table 1 for full genotypes)*.* Flies with reduced *Ldh* expression in clock cells, neurons, or glia were obtained by crossing *tim^ts^*, *elav^ts^*, and *repo^ts^* females to UAS-*Ldh^RNAi^* (BDSC #33640) males. For controls, *tim^ts^*, *elav^ts^*, and *repo^ts^* females were crossed to *w^1118^* males. Additionally, *elav^ts^* females were crossed to males with the genomic insertion of the *Ldh^RNAi^* construct but lacking an RNAi coding sequence (BDSC #36303), which we call UAS-*RNAi^control^* (Supporting Information Supplementary Table 1). The progeny of these crosses were maintained in 18°C throughout development. Two to three days after eclosion, mated males were transferred to 25°C or 27°C as specified in results. Longevity was measured as described [48] for each genotype and temperature. Mortality curves were plotted using GraphPad Prism 6 (GraphPad Software Inc. San Diego, CA).

### Quantitative reverse transcription polymerase chain reaction (qRT-PCR)

Young and old, 5 and 55 days of age respectively, *w^1118^* flies were collected at 6-hour intervals over 24 hours in 12:12 LD. All other genotypes were collected at a single time point at the lights off transition (ZT12). Heads of 50 flies were separated using liquid nitrogen-cooled stainless steel sieves. Samples were homogenized in Trizol (Sigma-Aldrich, St Louis, Missouri) using a Kontes handheld pestle followed by rDNAse I (Takara, Japan) treatment, phenol/chloroform precipitation, and ethanol/sodium acetate precipitation as described [49]. Maxima First Strand cDNA synthesis kit (Thermo Scientific) was used to synthesize cDNA. Real-time PCR was performed with Power SYBR Green (Applied Biosystems) on a Step One Plus PCR system (Applied Biosystems). Primers had the following sequences: *Dcp2* forward 5’ CCAAGGGCAAGATCAATGAG 3’, *Dcp2* reverse 5’ GCATCGATTAGGTCCGTGAT 3’, *Ldh* forward 5’ CGTTTGGTCTGGAGTGAACA 3’, *Ldh* reverse 5’ GCAGCTCGTTCCACTTCTCT 3’. RNA levels were normalized to *Dcp2* and analyzed by the 2-ΔΔCT method.

### Analysis of neurodegeneration

Heads of 55-day old *elav^ts^>Ldh* and *elav^ts^>w*, or *repo^ts^>Ldh* and *repo^ts^>w* or 75-day old *elav^ts^>Ldh^RNAi^* and *elav^ts^>RNAi^control^* mated males were randomly placed side by side in a collar, processed as one sample, embedded in the same paraffin block, and cut into 7μm sections. After removing the paraffin, the sections were embedded in Permount and analyzed by an experimenter blinded to the genotype. Due to the eye pigment, tissues are fluorescent, resulting in the vacuoles appearing in black. After examining all sections from a single head, an image was taken from the section that shows the most severe vacuolization phenotype. Vacuoles were identified, counted, and their surface area measured using the magic wand tool in Photoshop before the genotype was revealed. For a detailed description of the method and analysis including a video see [50].

### Analysis of locomotor activity rhythms

Activity rhythms were measured as described [48, 49]. Briefly, 20-, 35-, and 50-day old males of the genotypes specified in the results section were placed in individual activity tubes and loaded into the TriKinetics *Drosophila* Activity Monitoring System (DAMS; TriKinetics, Waltham, MA). Data were collected with DAMS every 1 minute for three consecutive days in LD 12:12 followed by six days in constant darkness. Data were analyzed using Clocklab software (v.6.0.50, Actimetrics; Wilmette, IL) to generate actograms and periodograms. Chi-squared periodograms of individual flies were used to determine circadian rhythmic behavior in constant darkness. Flies with a periodogram amplitude peak near 24-hours breaking the 99% confidence line were deemed rhythmic. The average daily activity levels were calculated using the activity counts during the six consecutive days in constant darkness for each genotype.

### Western blots

Groups of 20 whole heads were lysed, homogenized, and sonicated in an extraction buffer containing 50mM Tris, 2% SDS, 1mM EDTA, and protease inhibitors. Protein extracts were quantified using a colorimetric assay for protein concentration (DC Protein Assay; Bio-Rad), resolved in a 10% SDS-PAGE, and blotted onto a PVDF membrane (Bio-Rad). Membranes were probed with mouse monoclonal anti-β-tubulin (1:1000) (E7; Developmental Studies Hybridoma Bank) and polyclonal rabbit anti-LDHA (1:1000) (PA5-26531; ThermoFisher). This LDHA antibody was chosen based on its reactivity to *Drosophila* LDH protein using flies overexpressing *Ldh* (Supporting Information Supplementary Figure 1). Subsequently, membranes were probed with the appropriate horseradish peroxidase-conjugated secondary antibodies, goat anti-mouse AP130P (1:1000; Millipore) or goat anti-rabbit AP307P (1.5:1000; Millipore). Blots were imaged using Pierce ECL Western blotting substrate (ThermoFisher) in a Bio-Rad Molecular Imager (ChemiDoc XRS; Bio-Rad).

### LDH enzymatic activity

LDH enzyme activity was determined by measuring the rate of interconversion between pyruvate and lactate and concurrent changes in the NAD^+^/NADH redox couple. Groups of 20-50 heads were lysed, homogenized, and sonicated in a buffer containing 100mM potassium phosphate, 2mM EDTA, and protease inhibitors. Protein concentration was determined using a colorimetric assay (DC Protein Assay), and 6μg of protein was loaded into 50μl reactions done in triplicate in a 96-well plate. The conversion of pyruvate to lactate results in the oxidation of NADH whereas the conversion of lactate to pyruvate results in the reduction of NAD^+^. Changes in NADH fluorescence were detected using a Tecan M1000 microplate reader with excitation/emission wavelengths of 340/460nm then plotted on a standard curve generated with known concentrations of NADH to determine NADH concentration at each time point. Pyruvate to lactate activity (decrease in NADH concentration over time) was measured in a reaction containing 1M sodium pyruvate (Sigma) and 6.4mM NADH (Sigma) in a 500mM potassium phosphate buffer. Lactate to pyruvate activity (increase in NADH concentration over time) was measured in a reaction containing 1M sodium lactate, 14.3mM NAD^+^, and 0.52mM hydrazine in a buffer containing 0.5M glycine and 2.5mM EDTA. Enzyme activity is reported in international units (U), as defined by the conversion of 1μmol of NADH/minute/mg protein.

### Polar metabolite extraction and 1D ^1^H nuclear magnetic resonance (NMR)

Polar metabolites were extracted from the heads of 5- and 55-day old males using a modified 1:1:1 methanol/water/chloroform protocol [51, 52]. Groups of 200 flies were flash-frozen in liquid nitrogen; heads were separated in liquid nitrogen, lyophilized for 48 hours, and the dry weight of each sample was recorded. All reagents used in the following steps were chilled and kept on ice. Heads were pulverized and then homogenized in methanol/water mixture using a bead beater (Qiagen) with 5-7 ZR BashingBeads (Zymo Research). The remaining water and chloroform were added sequentially, followed by agitation and incubation on ice. Samples were centrifuged at 2000g for 5 minutes to separate the phases. The polar layer was transferred to new tubes, dried overnight in a speed-vac, and placed at -80°C. Pellets were rehydrated in 5mM sodium phosphate buffer with IS-2 Chenomx Internal Standard with DSS-d6 (Chenomx; Canada). Samples were loaded into 3mm NMR tubes (Bruker; Switzerland). NMR data were collected on an 800 MHz (18.8 T) Bruker Avance III HD NMR spectrometer (Bruker; Switzerland) equipped with a triple resonance cryogenic probe. 1D NOESY spectra with solvent pre-saturation were acquired at 25°C with a spectral window of 9615.385 Hz (12 ppm), the acquisition time of 4 seconds, recycle delay of 1 second, calibrated 90-degree pulse and 512 scans. The total acquisition time for each sample was ~45 minutes. Data were apodized with 0.5Hz exponential line broadening, zero-filled to twice the number of points, Fourier Transformed and phased using the Chenomx NMR Suite Processor program (Chenomx; Canada). Metabolites were identified and quantified using the Chenomx NMR Suite Profiler. Metabolite concentrations were normalized by the dry weight of the sample.

## Supplementary Material

Supplementary Figures

Supplementary Tables
