# Supplementary material for: Lactate dehydrogenase expression modulates longevity and neurodegeneration in Drosophila melanogaster
Source: Aging (Albany NY). 2020 Jun 2;12(11):10041–58. doi: 10.18632/aging.103373 (PMC7346061; doi:10.18632/aging.103373)
Supplement: Supplementary Figures [file aging-12-103373-s002..pdf]

SUPPLEMENTARY FIGURES

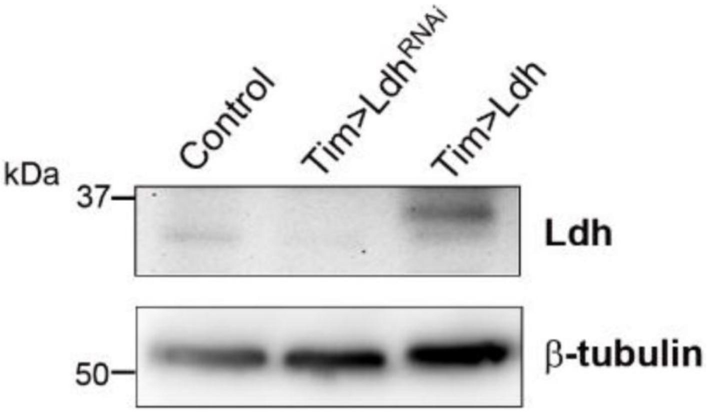

Supplementary Figure 1. LDH antibody validation. Western blot analysis of head extracts from 10-day-old flies overexpressing *Ldh* in all circadian clock cells (*tim>Ldh*), flies with *Ldh* downregulated by expression of an RNAi construct (*tim>Ldh*^{RNAi}), and control *tim>w* flies. The higher molecular weight of LDH in *tim>Ldh* flies, compared to endogenous LDH in control flies, is attributed to an incorporated triple HA tag within the *Ldh* transgene (Supporting Information Supplementary Table 1).

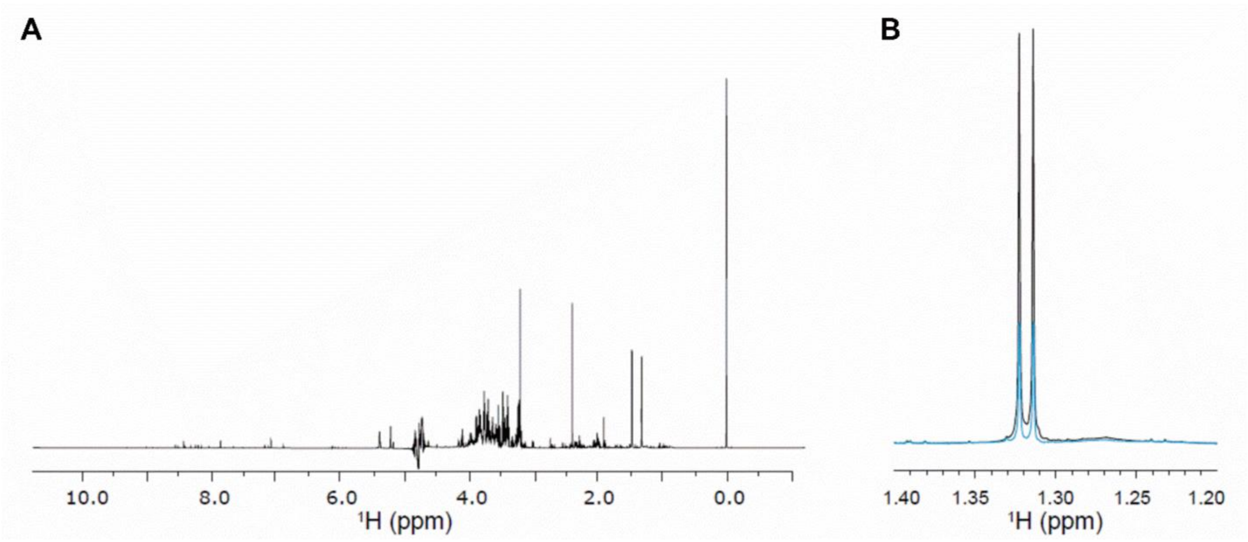

Supplementary Figure 2. Representative NMR spectrum data. (**A**) Representative 1D 1H NMR spectrum of young male fly heads collected at ZT12. (**B**) Representative overlay of NMR spectra obtained from the heads of young (light blue) versus old (dark blue) flies collected at ZT12. The region shown (1.2 to 1.4 ppm) contains the strong doublet for the lactate methyl group. Spectra were normalized to the DSS internal standard peak for comparison.
