# Supplementary material for: Lactate dehydrogenase expression modulates longevity and neurodegeneration in Drosophila melanogaster
Source: Aging (Albany NY). 2020 Jun 2;12(11):10041–58. doi: 10.18632/aging.103373 (PMC7346061; doi:10.18632/aging.103373)
Supplement: Supplementary Tables [file aging-12-103373-s001..pdf]

SUPPLEMENTARY TABLES

Supplementary Table 1. Names and genotypes of the flies used in this study.

Name used	Genotype
tim>Ldh	*w*;*tim*-GAL4/+;{UAS-*ImpL3*.ORF.3xHA.GW}
tim>w	w;tim-GAL4/+;+/+
tim^{ts}*>Ldh*	*yw*;*tim*-GAL4/+;*tub*-GAL80^{ts}/{UAS-*ImpL3*.ORF.3xHA.GW}
tim^{ts}*>w*	*yw*;*tim*-GAL4/+;*tub*-GAL80^{ts}/+
elav^{ts}*>Ldh*	;*elav*-GAL4/+;*tub*-GAL80^{ts}/{UAS-*ImpL3*.ORF.3xHA.GW}
elav^{ts}*>w*	;*elav*-GAL4/+;*tub*-GAL80^{ts}/+
repo^{ts}*>Ldh*	;*tub*-GAL80^{ts}/+;*repo*-GAL4/{UAS-*ImpL3*.ORF.3xHA.GW}
repo^{ts}*>w*	;*tub*-GAL80^{ts}/+;*repo*-GAL4/+
w>Ldh	*w*;;{UAS-*ImpL3*.ORF.3xHA.GW}
tim^{ts}*>Ldh*^{RNAi}	*yw*;*tim*-GAL4/+;*tub*-GAL80^{ts}/P{y+ v+ UAS-Ldh-RNAi}attP2/+
tim^{ts}*>w*	*yw*;*tim*-GAL4/+;*tub*-GAL80^{ts}/+
elav^{ts}*>Ldh*^{RNAi}	;*elav*-GAL4/+;*tub*-GAL80^{ts}/P{y+ v+ UAS-Ldh-RNAi}attP2
elav^{ts}*>w*	;*elav*-GAL4/+;*tub*-GAL80^{ts}/+
elav^{ts}*>RNAi*^{control}	;*elav*-GAL4/+;*tub*-GAL80^{ts}/P{y+CaryP}attP2
repo^{ts}*>Ldh*^{RNAi}	;*tub*-GAL80^{ts}/+;*repo*-GAL4/P{y+ v+ UAS-Ldh-RNAi}attP2
repo^{ts}*>w*	;*tub*-GAL80^{ts}/+;*repo*-GAL4/+
w>Ldh^{RNAi}	*w*;;P{y+ v+ UAS-Ldh-RNAi}attP2

Supplementary Table 2. Longevity data of *Ldh* overexpressing flies and controls.

Temperature		Genotype	N	Lifespan (Days)		Gehan-Breslow-Wilcoxon p-value
				Median	Max	
18°C - 27°C		*tim*^{ts}*>Ldh*	104	49	63	-
		tim^{ts}*>w*	86	66.5	77	< 0.0001
		elav^{ts}*>Ldh*	99	63	85	-
		elav^{ts}*>w*	94	71	82	0.0129
		repo^{ts}*>Ldh*	99	63	82	-
		repo^{ts}*>w*	95	69	76	0.0124
		w>Ldh	102	79	90	< 0.0001*
18°C - 25°C		*tim*^{ts}*>Ldh*	96	67	79	-
		tim^{ts}*>w*	84	79	95	< 0.0001
	BR1	*elav*^{ts}*>Ldh*	97	79	107	-
		elav^{ts}*>w*	98	85.5	102	0.0022
	BR2	*elav*^{ts}*>Ldh*	180	74	102	-
		elav^{ts}*>w*	176	85	110	< 0.0001
		repo^{ts}*>Ldh*	99	79	97	-
		repo^{ts}*>w*	101	85	102	< 0.0001
		w>Ldh	97	94	107	< 0.0001*

*p-values of this control genotype are in comparison to all genotypes with overexpressed *Ldh*.

Supplementary Table 3. Longevity data of *Ldh*^{RNAi} expressing flies and controls.

Temperature		Genotype	N	Lifespan (Days)		Gehan-Breslow-Wilcoxon p-value
				Median	Max	
18°C - 27°C		tim^{ts}>Ldh^{RNAi}	81	77	90	-
		tim^{ts}>w	86	66.5	77	< 0.0001
		$elav^{ts}$>Ldh^{RNAi}	97	84	94	-
		$elav^{ts}$>w	94	71	82	< 0.0001
		$repo^{ts}$>Ldh^{RNAi}	93	76	84	-
		$repo^{ts}$>w	95	69	76	< 0.0001
		w>Ldh^{RNAi}	97	73	90	< 0.0001*
18°C - 25°C		tim^{ts}>Ldh^{RNAi}	97	88	106	-
		tim^{ts}>w	84	79	95	< 0.0001
		$elav^{ts}$>Ldh^{RNAi}	97	104	114	-
		$elav^{ts}$>w	98	85.5	102	< 0.0001
		$repo^{ts}$>Ldh^{RNAi}	104	87.5	106	-
		$repo^{ts}$>w	101	85	102	0.0287
		w>Ldh^{RNAi}	91	79	101	< 0.0001*
	BR1	$elav^{ts}$>Ldh^{RNAi}	187	98	112	-
		elav^{ts}>$\mathrm{RNAi}^{control}$	195	79	103	< 0.0001
	BR2	$elav^{ts}$>Ldh^{RNAi}	98	91	117	-
		elav^{ts}>$\mathrm{RNAi}^{control}$	124	78	114	< 0.0001

*p-values of this control genotype are in comparison to all genotypes expressing *Ldh*^{RNAi}.
